# The Influence of Wide-Directional Asymmetric Spraying on Machining Deformation of Aluminum Alloy Plates

**DOI:** 10.3390/ma18204802

**Published:** 2025-10-21

**Authors:** Yang Li, Zhongkun Lin, Yanan Li, Xiwu Li, Kai Zhu, Mingyang Yu, Ying Li, Hongwei Yan

**Affiliations:** 1 State Key Laboratory of Nonferrous Structural Materials, China GRINM Group Co., Ltd., Beijing 100088, China; gyyliyang@grinm.com (Y.L.); liyanan@grinm.com (Y.L.); lixiwu@grinm.com (X.L.); zhukai@grinm.com (K.Z.); yumingyang@grinm.com (M.Y.); liying@grinm.com (Y.L.); yanhongwei@grinm.com (H.Y.); 2 GRIMAT Engineering Institute Co., Ltd., Beijing 101407, China; 3 General Research Institute for Nonferrous Metals, Beijing 100088, China

**Keywords:** aluminum alloys, machining deformation, residual stress, asymmetric stress, aerospace components, spray quenching, finite element simulation, machining strategy, stress release, deformation control

## Abstract

This study investigates the machining deformation of thick aluminum alloy plates, specifically in aerospace frame components, focusing on the influence of asymmetric residual stress states and machining strategies. Aluminum alloys are commonly used for large structural components due to their strength, formability, and corrosion resistance. However, machining these components often leads to deformation caused by residual stress release, cutting forces, and thermal effects. Using finite element simulations and experimental validation, the study analyzes how asymmetric residual stresses, induced by spray quenching, affect deformation patterns during machining. It is found that lower initial stress asymmetry results in less deformation, while machining sequences that optimize stress release significantly reduce the final distortion. Among the strategies tested, the diagonal milling sequence yielded the smallest deformation, achieving a reduction of up to 4%. The study concludes that both the initial residual stress state and the machining strategy are critical in controlling deformation, offering insights for improving machining processes in aerospace manufacturing to enhance precision and reliability.

## 1. Introduction

Aluminum alloys have become a key material for large monolithic structural components (such as wing spars and fuselage frames) in the aerospace industry due to their excellent specific strength, formability, and corrosion resistance [1]. Large structural components manufactured from high-strength aluminum alloy thick plates via efficient CNC machining can significantly reduce structural weight and enhance load-bearing efficiency [2]. However, such components often experience uncontrolled deformation or even cracking during machining due to residual stress release, the coupled effects of cutting forces and heat, and improper material removal path planning [3]. This severely constrains machining accuracy and service reliability [4].

Machining deformation of thick plates primarily originates from the initial residual stresses within the blank [5]. To meet mechanical property requirements, aluminum alloy thick plates undergo solution-quenching heat treatment. The severe temperature gradients generated during this process induce residual stresses reaching up to several hundred megapascals within the plate [6]. Although processes like pre-stretching and pre-compression can partially reduce the stress levels, the release and redistribution of residual stresses during machining—where material removal rates often exceed 90%—still induce significant deformation [7].

Extensive research confirms that the initial residual stress state is a core parameter for predicting and controlling deformation [8]. Husson et al. successfully reduced machining deformation by 40% by optimizing the residual stress distribution in gearbox blanks through heat treatment [9]. Fu et al. proposed a piecewise numerical model enabling high-precision deformation prediction based on initial residual stress, and validated its effectiveness through finite element analysis and experiments [10]. However, existing studies predominantly assume a symmetric distribution of residual stresses through the thickness direction. In contrast, industrial blanks often exhibit stress asymmetry due to non-uniform cooling or rolling processes [11]. The influence mechanism of this asymmetry on deformation has not yet been systematically revealed [12].

Cutting parameters (cutting speed, feed rate, depth of cut) directly influence surface layer plastic deformation and the transient stress field by altering the cutting force/heat load [13]. On one hand, tool extrusion and friction lead to work-hardened layers and surface residual stresses [14]. On the other hand, the moving heat source effect (particularly prominent in high-speed milling) disrupts the initial stress equilibrium, exacerbating deformation [15]. Wu et al. optimized cutting parameter combinations for aerospace monolithic components through combined simulation and experimentation, achieving a 32% reduction in deformation magnitude [16]. Li et al. discovered that the depth of cut significantly affects the final deformation mode by altering the stress evolution path [17]. Although parameter optimization can locally suppress deformation, it is difficult to compensate for systematic deviations caused by deep-layer residual stress asymmetry [18].

The material removal path dictates the sequential release of residual stresses [19]. Rational strategies (e.g., symmetrical layer-by-layer cutting, adaptive toolpath direction) can suppress accumulated deformation through the “self-balancing effect” of stress release [20]. For instance, Chen et al. demonstrated that employing an inward-to-outward, alternately symmetrical milling sequence for frame-type structures can reduce sidewall warpage by 50% [21]. Regrettably, existing strategy research is largely confined to simple geometries [22]. There is a lack of dedicated path planning methods for aeronautical frame-type components with complex topologies (e.g., multi-rib thin-walled structures) that consider the coupling effect between stress asymmetry and structural stiffness [23].

Research on machining deformation behavior has predominantly focused on symmetric residual stress distributions, but real-world industrial blanks often display asymmetry due to irregular cooling or rolling processes. The impact of this stress asymmetry on deformation during machining is not well understood and remains underexplored. Existing studies generally assume a symmetric stress distribution, neglecting the complex effects of asymmetric residual stresses that arise in practical scenarios [24]. As a result, the ability to predict and control deformation accurately under asymmetric conditions remains a significant gap in current machining strategies [25,26,27].

To fill this gap, this study introduces asymmetric residual stress distributions in thick aluminum alloy plates by utilizing asymmetric spray-controlled quenching experiments and a multi-physics coupled finite element model. This research aims to quantify the relationship between spray parameters and stress asymmetry, shedding light on how the interaction between asymmetric residual stress and material removal strategy influences deformation behavior. Furthermore, a tailored deformation suppression strategy for frame-type aeronautical components is proposed, providing theoretical guidance for high-precision manufacturing in the aerospace industry.

## 2. Materials and Methods

### 2.1. Finite Element Simulation

#### 2.1.1. Simulated Hypothesis

To enhance computational efficiency and enable tractable modeling of the intricate thermal-fluid phenomena inherent in aluminum alloy spray quenching, key simplifications were implemented within the numerical framework [28]. These focus on mitigating complexities associated with heat transfer prediction, stress evolution, and distortion analysis: (1) Microstructural Homogeneity: The computational model treats the thick plate as a macroscopically isotropic continuum, characterized by uniform composition and microstructure throughout its volume, devoid of preferential crystallographic orientation [29]. (2) Pre-Quenching Stress State: Internal residual stresses within the plate prior to quenching are considered effectively eliminated [30]. This simplification is grounded in the metallurgical rationale that the preceding extended solution treatment at elevated temperatures facilitates near-complete stress relaxation [31]. (3) Phase Transformation Effects: This simplification enables the model to focus on the dominant thermomechanical effects during quenching and machining processes, albeit with a potential minor underestimation of transient stress states. It should be noted that the core objective of solution treatment is to achieve a supersaturated solid solution by fully dissolving the secondary phases. During the rapid cooling stage of spray quenching, the precipitation kinetics are generally weak [32]; based on this, the contribution of latent heat from phase transformations is excluded from the thermal analysis in this study [33].

#### 2.1.2. Model and Parameters of Simulation

The finite element analysis employed a computational domain measuring 320 mm in length, 180 mm in width, and 26 mm in thickness. A structured hexahedral mesh with uniform element dimensions of 2 × 2 × 1 mm was applied, resulting in a total of 374,400 discrete elements. Thermomechanical properties of AA7055 aluminum alloy critical to the simulation—including density, thermal conductivity, coefficient of linear thermal expansion, yield strength, specific heat capacity, and elastic modulus—are comprehensively detailed in Table 1. The thermal boundary conditions defined the quenching process with an initial uniform temperature field of 475 °C, cooling progressively to a final equilibrium state of 25 °C.

Accurate representation of interfacial heat transfer coefficients (HTC) constitutes a critical boundary parameter in quenching simulations. Prior research establishes that inverse methodology-derived HTC values enable precise prediction of transient thermal fields within quenched plates. This study implemented spatially averaged HTC values as thermal boundary conditions to model temperature evolution throughout the quenching process. Experimentally determined average HTC magnitudes corresponding to discrete spray water flow rates are compiled in Table 2. Flow rates were quantified in real-time using turbine flowmeters, confirming a maximum single-nozzle capacity of 0.64 m^3^/h. Systematic flow reduction trials subsequently established operating points at 0.48 m^3^/h, 0.32 m^3^/h, and 0.16 m^3^/h. For parametric consistency, these conditions were designated as normalized flow rates of 1.0, 0.75, 0.5, and 0.25, respectively.

**Figure 1:**
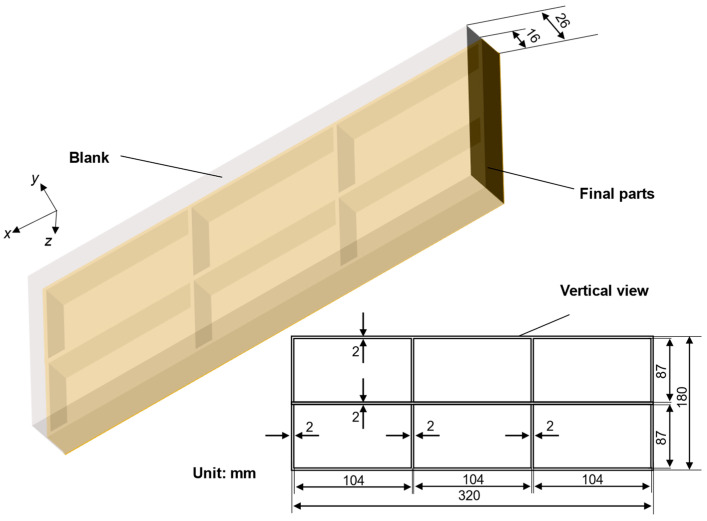
Geometric model of blank and final parts.

Thermomechanical analysis of the quenching process employed ANSYS-2023 R1 based sequential coupling methodology, where convective heat transfer solutions informed subsequent stress field simulations. To evaluate machining distortion in frame components, the element deactivation/reactivation technique was implemented, simulating progressive material removal. The representative monolithic structure (illustrated in Figure 1) features overall dimensions of 320 mm (length) × 180 mm (width) × 16 mm (height), with consistent 2-mm wall thicknesses throughout its frame architecture.

### 2.2. Experimental Section

#### 2.2.1. Materials for Experimentation

Experimental validation utilized AA7055 aluminum alloy thick plates (geometric specifications matching the finite element model; chemical composition shows in Table 3). Recognizing that surface topography influences quenching heat transfer and consequently residual stress homogeneity, all spray-exposed specimen surfaces were precision-ground to achieve uniform arithmetic mean roughness (Ra) of 0.8 μm prior to testing.

#### 2.2.2. Spray Quenching Experiment

**Figure 2:**
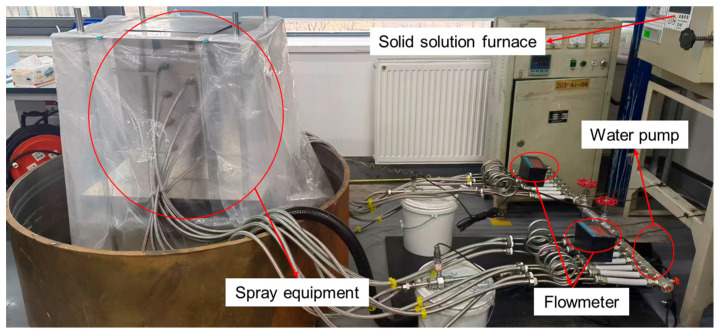
Machining experiment and deformation monitoring method.

Heating elements, modular spray nozzles, and a closed-loop water circulation unit were integrated into the quenching system, with the key components and layout illustrated in Figure 2. The hydraulic circuit comprises a reservoir, frequency-controlled centrifugal pump (PRODN CHM4-2DC, Manufacturer: Wenzhou Qiyuan Pump Valve Manufacturing Co., Ltd.; Place of Origin: Wenzhou, China), precision valves, particulate filters, turbine flowmeters, and corrosion-resistant stainless steel conduits. Circulating water temperature was regulated at 25 °C with ±1 °C tolerance. System pressure and volumetric flow were modulated via pump frequency adjustment and independently verified using calibrated flowmeters. Specimens underwent solution treatment at 475 °C for 4 h in a forced-convection furnace to ensure thermal homogeneity and residual stress annihilation. Solution-treated samples were then rapidly transferred to the quenching station (<10 s transition time), followed by immediate valve activation. Spray quenching duration exceeded 120 s for complete thermal stabilization.

#### 2.2.3. Machining Experiment and Characterization Method of Deformation

**Figure 3:**
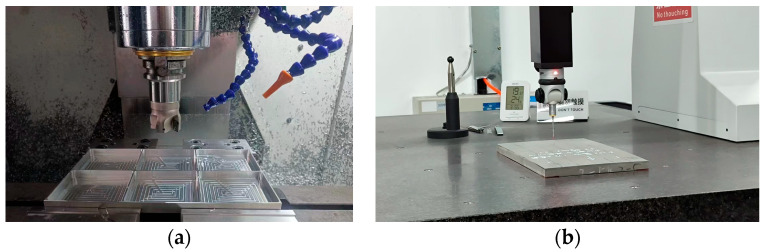
Equipment diagram: (**a**) CNC milling machine; (**b**) EXPLORER 06.08.06.

Experimental validation of the simulated distortion behavior under asymmetric stress conditions and part-flipping strategies was conducted via CNC machining trials (Figure 3a, Manufacturer: Shenyang Machine Tool (Group) Co., Ltd.; Place of Origin: Shenyang, China), with the corresponding machining parameters detailed in Table 4. During the trials, tool wear was monitored periodically, and surface roughness (Ra) was maintained below 1.6 μm through regular tool replacement to ensure consistent machining quality.

Post-machining dimensional verification was performed using a computer-controlled coordinate measuring machine (CMM, model: EXPLORER 06.08.06; Figure 3b, Manufacturer: Wenzhao Precision Measurement Technology Co., Ltd.; Place of Origin: Hangzhou, China). To guarantee metrological accuracy and traceability, all measurements were carried out in a temperature-controlled laboratory (20 ± 1 °C) after the workpiece achieved thermal stabilization. The CMM was equipped with a touch-trigger probe featuring a 2 mm ruby stylus, and probing paths were pre-planned to avoid collision and ensure measurement repeatability. For this CMM, the Maximum Permissible Error (MPE) is ± (1.8 + 3L/1000) μm; additionally, a Gage R&R study was conducted, yielding a %GRR of 8.2%—a value well within acceptable limits for dimensional measurement.

## 3. Results and Discussions

### 3.1. Residual Stress Distribution Under Asymmetric Spray Conditions

**Figure 4:**
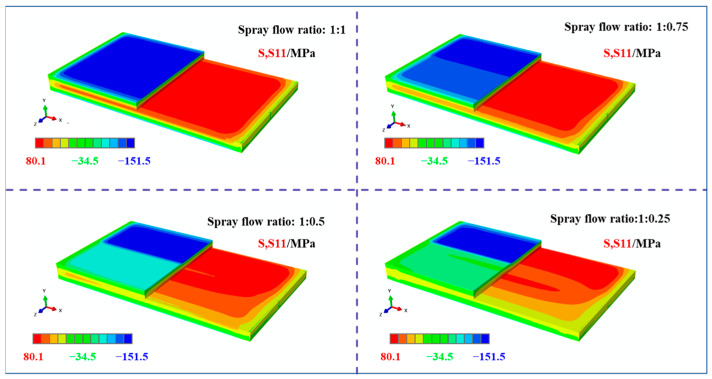
Stress map of the plate under different spraying conditions (S11 direction, specific dimensions shows in Figure 1).

By setting different simulation parameters, the distribution of residual stresses within a plate subjected to asymmetric spray cooling along the width direction was numerically investigated. Figure 4 shows the stress contour map of the entire plate along the longitudinal direction (S11 direction). As can be observed from the residual stress distribution, after spray cooling, the surface of the plate exhibited compressive stresses, whereas the core region showed tensile stresses. The maximum stresses were located in the high spray flow rate regions, with transitional zones present between regions with different spray intensities. The magnitudes of these transitional zones increased with increasing disparity in the spray flow rates. Additionally, the stress concentrations at the sharp edges and corners were notably reduced.

**Figure 5:**
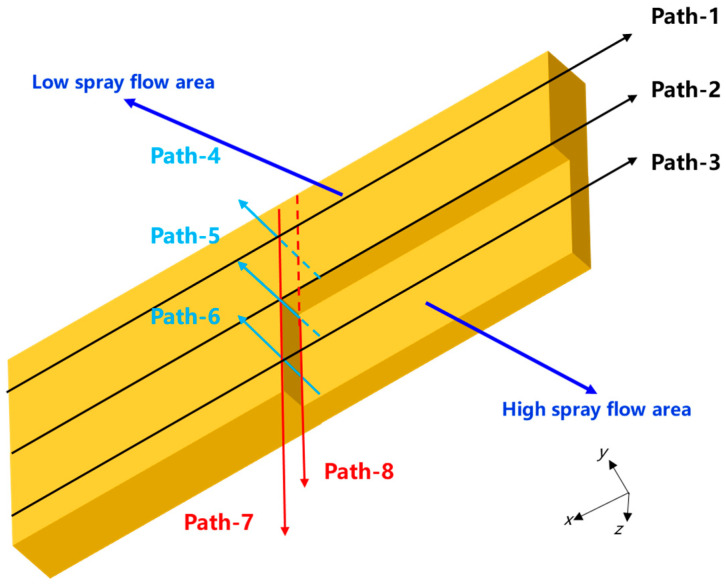
Schematic representation of the pathway.

Overall, the distribution pattern of the residual stress within the plate subjected to asymmetric spraying along the width direction was rather intricate. To gain a clear insight into the overall stress distribution characteristics of the plate, eight distinct paths were designed to characterize its overall stress level. First, three paths located at 1/4, 1/2, and 3/4 of the width on the bottom surface along the length direction were selected for surface stress characterization, denoted as Path-1, Path-2, and Path-3, respectively. Second, three paths at 1/4, 1/2, and 3/4 of the width along the thickness direction were employed for inner stress characterization, labeled as Path-4, Path-5, and Path-6, respectively. Finally, paths were arranged on the surface and at the mid-thickness along the width direction to characterize the stress in the width direction, referred to as Path-7 and Path-8, respectively. A schematic of the paths is presented in Figure 5.

**Figure 6:**
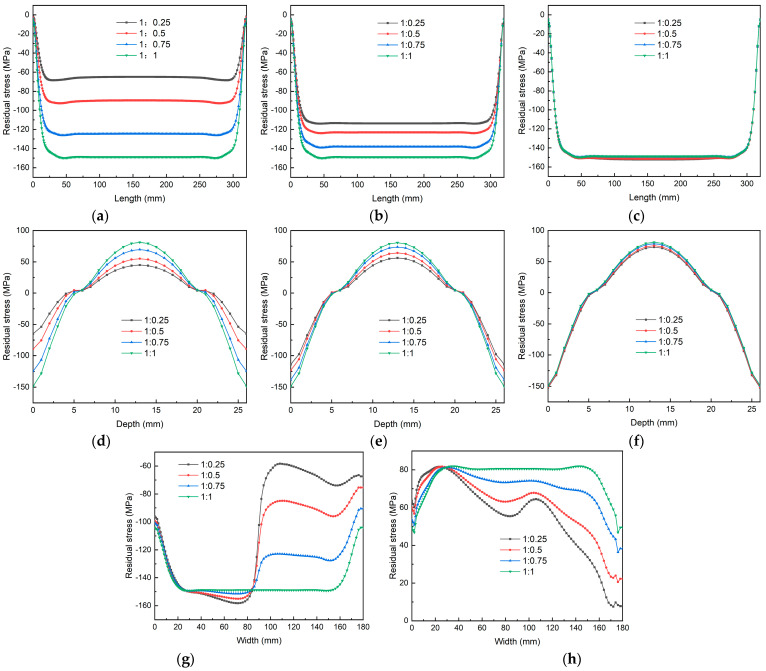
Stress characterization results of different spray flows under different paths (S11 direction): (**a**) Path-1; (**b**) Path-2; (**c**) Path-3; (**d**) Path-4; (**e**) Path-5; (**f**) Path-6; (**g**) Path-7; (**h**) Path-8.

Figure 6 shows the stress characterization results for the plates under the four stress states along different paths. From Figure 6a–c (i.e., the stress characterization results of the plates along Path-1, Path-2, and Path-3), it can be observed that the surface stress of the plates is higher on the side with a high spray flow rate and lower on the side with a low spray flow rate. The overall stress level of the plates gradually decreased as the spray flow rate on one side decreased, although the magnitude of the reduction varied. At low spray flow rates, the surface stress of the plates decreased significantly with a gradual reduction in the spray flow rate. When the spray flow rate on one side decreased from 1 to 0.25, the surface stress along Path-1 decreased significantly, with the peak compressive stress dropping from 152 MPa to 64.9 MPa. In the middle part along the width direction, the surface stress level of the plates also decreased with a reduction in the spray flow rate. When the spray flow rate on one side decreased from 1 to 0.25, the peak compressive stress on the surface along Path-2 decreased from 152 to 113.8 MPa, with a significantly smaller variation amplitude. On the side with a high spray flow rate, the surface stress level of the plates remained essentially unchanged as the spray flow rate on the other side decreased.

As illustrated in Figure 6d–f (i.e., the stress characterization results of the plate along Path-4, Path-5, and Path-6), the overall stress level of the plate in the thickness direction exhibited a decreasing trend with the reduction in spray flow rate on one side. On the side with a low spray flow rate, the peak compressive stress in the core of the plate decreased significantly as the spray flow rate was gradually reduced. When the spray flow rate on one side was decreased from 1 to 0.25, the peak compressive stress in the core along Path-4 decreased from 81.1 MPa to 55.0 MPa. In the middle portion along the width direction, the peak compressive stress in the core of the plate also decreased with a reduction in the spray flow rate. When the spray flow rate on one side was decreased from 1 to 0.25, the peak compressive stress in the core along Path-2 decreased from 81.1 MPa to 56.3 MPa, with a significantly smaller variation amplitude. On the side with high spray flow rate, the surface stress level of the plate undergoes only a slight change as the spray flow rate on the other side is reduced.

It can be observed from Figure 6g,h (i.e., the stress characterization results of the plate along Path-7 and Path-8) that both the surface compressive stress and core tensile stress of the plate along Path-7 and Path-8 have certain uniform regions. The smaller the difference in the spray flow rates, the larger the uniform region of the overall stress level of the plate and the shorter the transition zone from the low-spray-flow-rate area to the high-spray-flow-rate area. However, as the spray flow rate gradually increased, the transition zone between the two expanded, and the variation in the stress level within the transition zone became more drastic.

**Figure 7:**
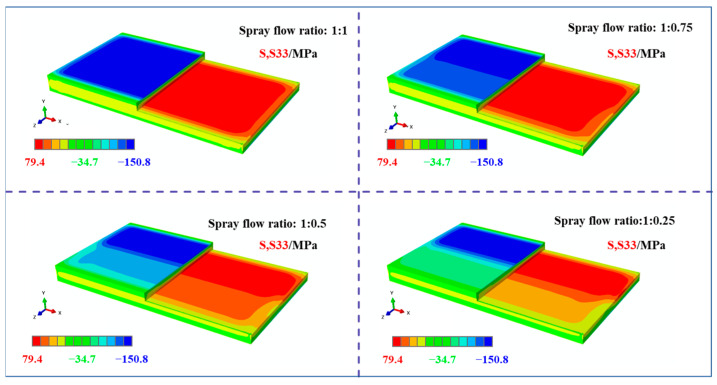
Stress maps of the plate under different spraying conditions (S33 direction; specific dimensions shown in Figure 1).

Figure 7 illustrates the stress contour map of the plate along the width direction (S33 direction). The results indicate that the stress distribution along the S33 direction exhibits a classic “compressive outer layer, tensile inner layer” pattern. The surface stresses along the longitudinal direction remained relatively uniform in both the low- and high-spray-flow-rate regions. However, as the spray flow rate decreased, the stress level on the low-flow side gradually diminished, leading to an increasing disparity between the two sides. This variation trend is consistent with that observed in the S11 direction and will not be discussed further.

### 3.2. Influence of Initial Residual Stress State on Machining Deformation

#### 3.2.1. Stress Results of Machined Frame Parts Under Different Initial Stress Conditions

To investigate the influence of initial residual stress states on machining-induced deformation of plates, numerical simulations were performed using the “element birth-and-death method” for four distinct initial stress conditions.

**Figure 8:**
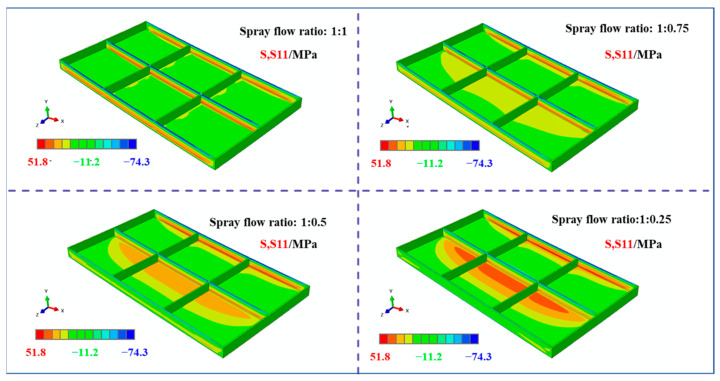
Stress maps of machined frame parts under different initial stress conditions (S11 direction, specific dimensions shows in Figure 1).

Figure 8 presents the stress contour maps along the longitudinal direction (S11) of the frame components machined from plates with different initial stress states under the same machining strategy. The analysis of these results revealed consistent features in the overall stress distribution. First, compressive stresses in the S11 direction were predominantly concentrated at the top of the longitudinal stiffeners, with lower magnitudes observed near the low- and high-spray-flow regions. Second, the tensile stresses in the S11 direction were primarily distributed in the core of the longitudinal stiffeners, also exhibiting lower levels near the low-spray-flow region. Notably, the S11 stress distribution varied significantly with different initial stress states. For uniformly sprayed plates, the stress distribution at the bottom of the frame component was approximately symmetric along the width direction and dominated by compressive stresses. However, as the stress level on one side decreased, the compressive stresses at the bottom gradually diminished on the low-spray-flow side. When the spray flow ratio reached 1:0.25, the stress state on this side transitioned from compressive to tensile.

**Figure 9:**
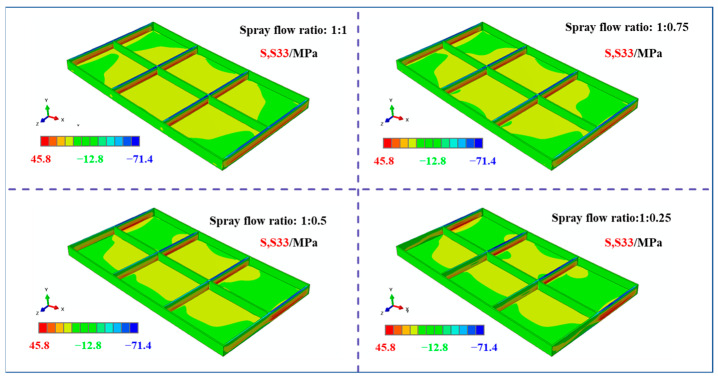
Stress map of machined frame parts under different initial stress conditions (S33 direction, specific dimensions shown in Figure 1).

Figure 9 illustrates the stress contour maps along the width direction (S33) of the frame components under identical machining conditions. It can be observed that the compressive stresses in the S33 direction are concentrated at the top of the transverse stiffeners, whereas the tensile stresses are localized in their core regions. Similar to the S11 stress distribution, the S33 stress distribution exhibited distinct variations with the initial stress states, following a consistent evolutionary trend: the compressive stresses at the bottom of the frame component gradually decreased on the low-spray-flow side as the stress level on one side diminished.

#### 3.2.2. Results of Internal Displacement Field of Machined Frame Parts Under Different Initial Stresses

**Figure 10:**
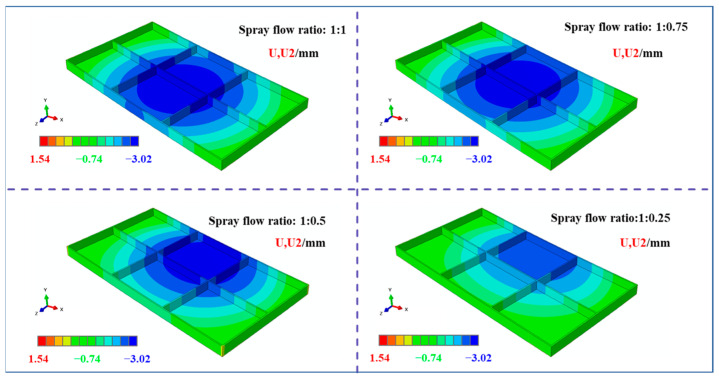
Clouds of displacement field after machining of plates with different initial stress states (U2 direction, specific dimensions shown in Figure 1).

As shown in Figure 10 (displacement field contour maps in the U2 direction), the deformation pattern of the plates under all four initial stress states was characterized by concave deformation at the bottom of the frame component. With decreasing stress level on one side, the location of maximum deformation gradually shifts toward the high-stress region.

**Figure 11:**
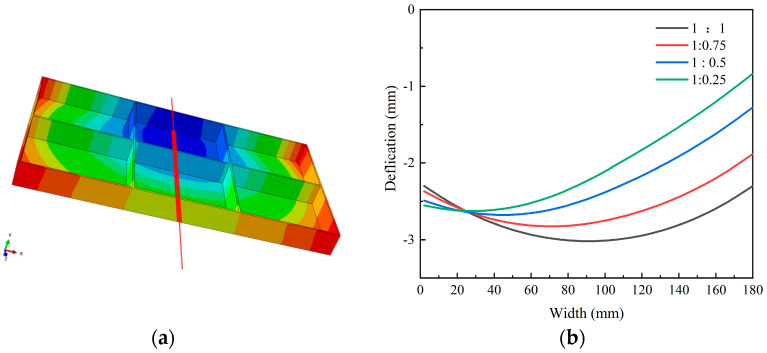
(**a**) Schematic diagram of deformation characterization path (path-9, specific dimensions shows in Figure 1); (**b**) Deformation characterization results of plates in different initial stress states after processing along path-9 (U2 direction).

To quantitatively characterize the machining-induced deformation, displacement measurements were conducted along Path-9 at the component bottom (schematic in Figure 11a), and the results are presented in Figure 11b. For the 1:1 spray flow ratio, the deformation was symmetric along the width direction, with maximum and minimum values of 1.18 mm and 0.91 mm, respectively. As the stress level on one side decreased, the overall deformation magnitude diminished, and the position of the maximum deformation shifted toward the high-stress side. However, at approximately 5/6 of the width, the deformation increased with decreasing stress on one side. This phenomenon can be attributed to the release of residual stress during material removal; to balance the asymmetric stress release, enhanced deformation occurs on the opposite side to maintain overall stress equilibrium.

#### 3.2.3. Machining Experiment Verification

**Figure 12:**
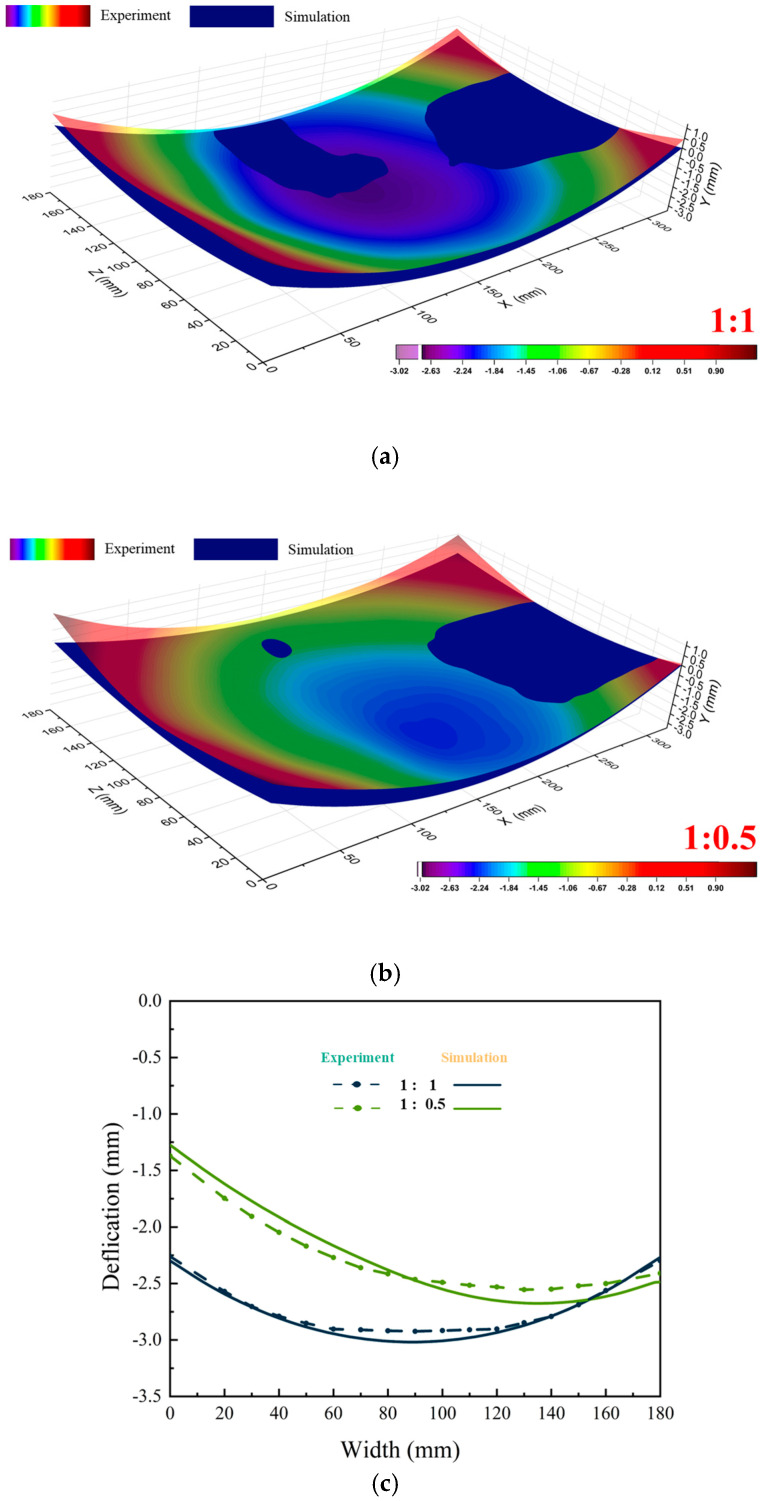
Comparison of experiments and simulation result: (**a**) 1:1; (**b**) 1:0.5; (**c**) Deformation characterization results of plates in different initial stress states after processing along path-9 (U2 direction).

Machining experiments were conducted to validate the simulation results. Plates with spray flow ratios of 1:1 and 1:0.5 were selected for verification, thewith experimental results are shown in Figure 12. For the 1:1 ratio, the maximum simulated deformation was 3.018 mm, compared to 2.924 mm in the experiments, resulting in a difference of 0.09 mm, indicating good overall agreement. For the 1:0.5 ratio, the maximum simulated deformation was 2.667 mm versus 2.544 mm experimentally, with discrepancies of 0.123 mm at the respective measurement points. Both cases exhibited significant deformation deviations, with consistent deformation trends between the simulations and experiments, confirming the accuracy of the numerical model.

### 3.3. Influence of Machining Strategy on Machining Deformation

#### 3.3.1. Machining Strategies

**Figure 13:**
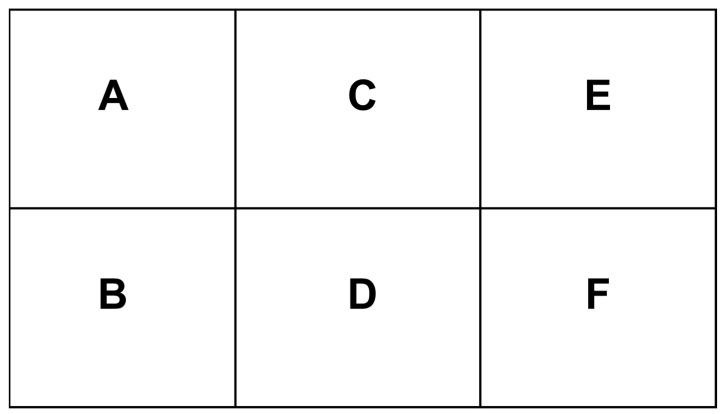
Diagram of machining strategies.

For plates with asymmetric residual stress distributions along the width direction, the influence of different machining sequences on stress evolution and deformation behavior was investigated. The six frames of the component are numbered as shown in Figure 13. Based on the actual production conditions and the asymmetric stress distribution, six machining sequences were designed: sequential milling (A-C-E-B-D-F, B-D-F-A-C-E), diagonal milling (A-F-E-B-C-D, F-A-B-E-D-C), and odd-even diagonal milling (A-D-C-F-E-B, F-C-D-A-B-E). The analysis focused on the results from the 1:0.5 spray flow ratio plates.

#### 3.3.2. Stress Results of Machined Frame Parts Under Different Machining Strategies

**Figure 14:**
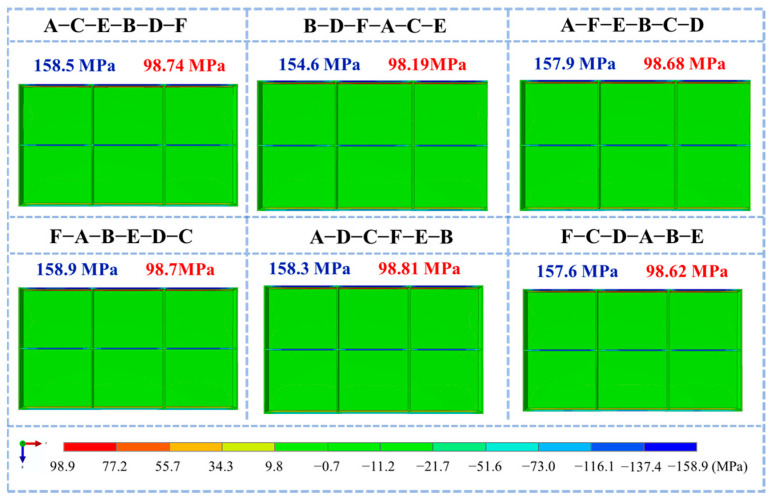
Stress maps of machined frame parts under different machining strategies. (S11 direction, specific dimensions shows in Figure 1).

Figure 14 presents the final S11 stress contour maps for the different machining sequences. Although the overall stress distribution patterns showed minor differences, compressive stresses were consistently concentrated at the top of the stiffeners and frame walls, with tensile stresses in the core regions of the frame walls. The S11 compressive stresses were primarily located at the top of the rolling-direction frame walls, with tensile stresses distributed along the frame walls. Variations in the residual stress magnitudes were observed between the sequences: B-D-F-A-C-E sequential milling resulted in the lowest peak tensile and compressive stresses; F-A-B-E-D-C diagonal milling produced the highest peak compressive stress; and A-D-C-F-E-B odd-even diagonal milling yielded the highest peak tensile stress. These findings indicate that the stress release sequence during machining significantly affects the final stress state of the frame components.

#### 3.3.3. Results of Internal Displacement Field of Machined Frame Parts Under Different Machining Strategies

**Figure 15:**
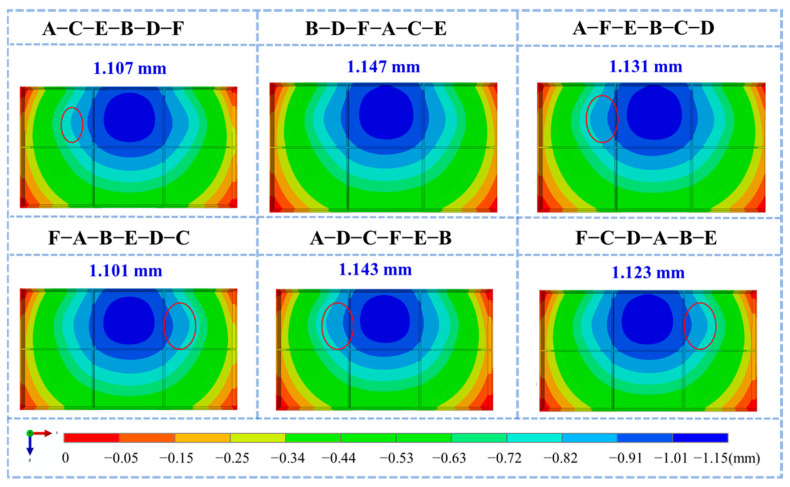
Displacement field cloud of frame parts under different machining strategies (U2 direction).

**Figure 16:**
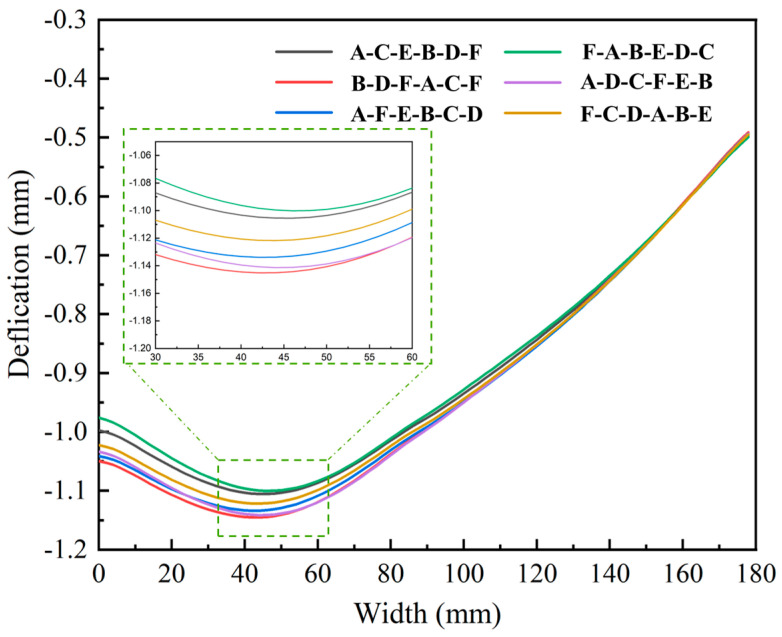
Deformation results of the bottom of structural components under different machining strategies.

Deformation contour maps and bottom deformation characterization results for different machining sequences are shown in Figure 15 and Figure 16, respectively. Although the overall deformation patterns were consistent, local variations existed (marked by red circles) owing to differences in the stress release sequences induced by the varying machining orders. The diagonal milling sequence F-A-B-E-D-C resulted in the smallest overall deformation (1.101 mm), whereas the sequential milling sequence B-D-F-A-C-E produced the largest deformation (1.147 mm). Optimizing the machining sequence achieved a 4% reduction in the maximum machining deformation.

**Figure 17:**
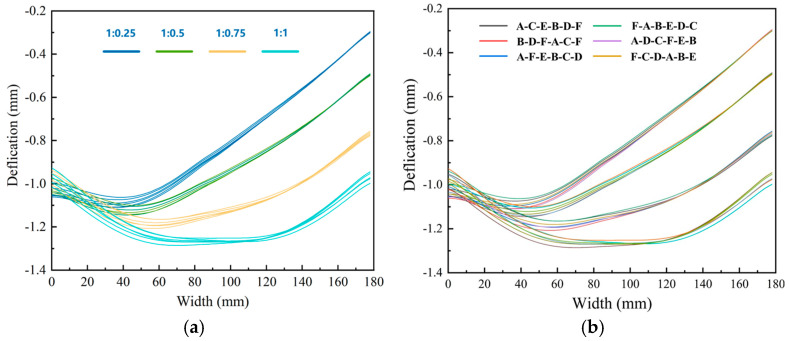
Comparison of deformation of frame parts under different initial stress states after different machining strategies: (**a**) Different initial stress; (**b**) Different machining strategies.

Figure 17 illustrates the combined effects of the initial stress states and machining sequences on the bottom deformation. Although the deformation patterns exhibited slight variations with the initial stress states, the diagonal milling sequence F-A-B-E-D-C consistently yielded optimal results. Additionally, the deformation magnitude decreased with lower initial stress levels, and this reduction was more pronounced on the lower-stress side as the stress asymmetry increased.

## 4. Conclusions

This study employed finite element simulations to generate plates with asymmetric residual stress distributions along the width direction and systematically investigated the influence of different initial stress states and machining strategies on machining-induced distortion. The principal conclusions are as follows.

(1) Influence of the Initial Stress State: Lower initial stress asymmetry consistently resulted in reduced post-machining stress levels and diminished overall deformation in the frame components. Deformation in the bottom region (U2 direction) predominantly exhibited a concave pattern. As the stress level on one side progressively decreased, the total deformation magnitude of the frame decreased correspondingly, and the location of the maximum bottom deformation shifted towards the side retaining higher stress.
(2) Machining Sequence Impact: While fundamental deformation patterns remained consistent across different machining sequences, the specific sequence governing the residual stress release during material removal significantly altered the final distortion state. Among the strategies evaluated, the Diagonal Milling sequence F-A-B-E-D-C yielded the minimal overall deformation, achieving a magnitude as low as 1.101 mm.
(3) Coupled Stress-State and Strategy Effects: Deformation patterns exhibited subtle variations depending on the initial stress state. Crucially, the magnitude of the initial stress asymmetry had a pronounced influence, with higher initial stress asymmetry directly correlating with increased final deformation in the assembled structure. The Diagonal Milling sequence F-A-B-E-D-C consistently demonstrated optimal performance across the evaluated stress states.

## Figures and Tables

**Table 1:** Parameters of AA7055 aluminum alloy.

Temperature (°C)	ρ (kg·m^−3^)	λ (W·m^−1^·°C^−1^)	α (10^−6^·°C^−1^)	c (J·kg^−1^∙K^−1^)	*R_p0.2_* (MPa)	*E* (GPa)
20	2841	145	22.7	913	266	73
100	2828	152	24	983	223	61
200	2807	160	24.2	1025	154	56
300	2787	167	25.2	1113	73	38
400	2761	171	26	1292	23	32
500	2735	178	27.5	1158	10	25

**Table 2:** Spray flow and heat transfer coefficient corresponding to standardized flow rate.

Normalized Flow Rates	$\mathrm{Water}\;\mathrm{Flow}\;\mathrm{Rate}\;(m^{3}\cdot h^{-1})$	$\mathrm{Heat}\;\mathrm{Transfer}\;\mathrm{Coefficients}\;(W\cdot m^{-2}\cdot {{}^{\circ}C}^{-1})$
1	0.64	10,000
0.75	0.48	8000
0.5	0.32	5500
0.25	0.16	4000

**Table 3:** Chemical composition of 7055 aluminum alloy (wt.%).

Zn	Cu	Mg	Zr	Fe	Si	Al
7.6~8.4	2.0~2.6	1.8~2.3	0.05~0.25	≤0.15	≤0.10	Bal.

**Table 4:** Machining parameters.

Rotation Speed (r·min^−1^)	Feed Speed (mm·min^−1^)	Axial Cutting Depth (mm)	Radial Cutting Width (mm)	Tool Inclination Angle	Tool Clearance Angle
1200	150	1	4	30°	8°

## Data Availability

The original contributions presented in this study are included in the article. Further inquiries can be directed to the corresponding author.
